# Health human resources challenges during COVID-19 pandemic; evidence of a qualitative study in a developing country

**DOI:** 10.1371/journal.pone.0262887

**Published:** 2022-01-24

**Authors:** Ali Reza Yusefi, Mehrdad Sharifi, Narjes sadat Nasabi, Esmat Rezabeigi Davarani, Peivand Bastani

**Affiliations:** 1 Department of Public Health, School of Health, Jiroft University of Medical Sciences, Jiroft, Iran; 2 Department of Emergency Medicine, School of Medicine, Shiraz University of Medical Sciences, Shiraz, Iran; 3 Development and Change Manager, Shiraz University of Medical Sciences, Shiraz, Iran; 4 Health in Disasters and Emergencies Research Center, Institute for Future Studies in Health, Kerman University of Medical Sciences, Kerman, Iran; 5 Health Human Resources Research Center, Shiraz University of Medical Sciences, Shiraz, Iran; The University of Hong Kong, HONG KONG

## Abstract

**Background:**

One of the main forthcoming challenges of healthcare systems against preparedness and management of the pandemic is the challenge of procurement and recruitment of the human resources. This study is aimed to explore the health human resources challenges during COVID-19 pandemic in Iran.

**Methods:**

This qualitative content analysis study was conducted in 2020. The study population includes all the Iranian human resources managers affiliated in Universities of Medical Sciences, hospitals and health centers managers and the health networks managers all over the country. 23 participants were included via purposeful sampling considering the inclusion criteria and were interviewed individually. After 23 semi-structured interviews, data were saturated. Then the data were analyzed through content analysis approach applying MAXQDA_10._

**Results:**

Three main themes of “organizational challenges”, “legal challenges”, and “personal challenges” were explored as the main challenges of health human resources management during COVID-19. On the one hand, organizational challenges include restricted financial resources, compensation discrimination, staffing distinction points, imbalance in the workload, weak organizational coordination, inefficient inter-sectoral relationships, parallel decisions, inefficient distribution of the human resources, lack of applied education, lack of integrated health protocols, lack of appropriate evaluation of performance, employee turnover, lack of clear approaches for staffing, and shortage of specialized manpower, and on the other hand, the personal challenges include insufficient knowledge of the employees, psychological disorders, reduction of self-confidence, burnout, workload increase, reduced level of job satisfaction, effects of colleague and patients bereavement and unsafety sense against the work place. Finally, the legal challenges that mostly related to the governments laws and regulations include lack of protocols for continuous supportive services, inappropriate approaches and instructions for teleworking, and lack of alternative plans and regulations for the human resources.

**Conclusion:**

Organizational, legal and personal challenges are among three main challenges of health human resources management during COVID-19 pandemic. Serious attention to these challenges should be considered by health policymakers in order to be prepared for facing new probable outbreaks and managing the present condition. The integrated comprehensive planning of human resources management for COVID-19 along with supportive packages for the personnel can be helpful.

## Background

COVID-19 has been emerged since December 2019 in China and less than four months, it has disseminated all over the world with an exponential growth. In such a condition, the World Health Organization (WHO) has announced it as a pandemic in 11 March 2020 [1, 2]. About the disease, it is obvious that, COVID-19 is an acute respiratory outbreak that is a close relationship with SARS corona virus [3]. According to the global statistics, the mortality rate was reported 3.4% [4]. In Iran, the first case of the disease was reported in 19 February 2020 in Qom and after a short time, it was disseminated to all other parts of the country [5]. In such a situation, one of the key elements of healthcare systems in prevention, control and treatment of the outbreaks the same as COVID-19 can be the health human resources [6].

Nowadays, the effects of socioeconomic, technological and cultural changes lead the healthcare systems to the continuous dynamic and more complexity [7]. One key factor of continuing success and goal achievement in these complex settings is the human resources [7]. Lack of specialized human resources in this area, their low quality knowledge and inappropriate distribution of their skills can be considered as a great obstacle in achieving the organizational development goals in the third millennium [8].

On the other hand, effective provision of health services is seriously affected by human resources. The main concerns in this area include inappropriate number, type, distribution method and the performance of the personnel in the health sectors [9]. In this regard, the optimal management of health human resources is considered as the significant responsibilities of the managers containing those activities for improving the level of competency and knowledge increase as well as developing the personnel`s skills [10]. Although the healthcare systems all over the world, increasingly face the challenges of shortages in the human resources and the inappropriate distribution of their skills [11], applying optimal management along with applied plans for quality improvement of these recourses can lead to improve the competencies as well as increasing the quality of services and decreasing the related challenges [12, 13].

The importance of optimal health human resource management can be considered from the WHO`s viewpoint. According to WHO report, a significant proportion of public governmental resources is allocated to healthcare sectors and the most fundamental part is related to health human resources [12–14]. A large amount of costs attributed to health human resources is related to the hospitals and health centers. According to the previous results of the studies in Iran, the costs of human resources is considered as 55–60% of the whole current expenditures of the hospitals and health centers [15–17]. This evidence highly shows the status and importance of optimal human resources management for healthcare sectors. In this regard, identification of the growth and improvement barriers of the personnel can be noticed as the first step [18].

COVID-19 pandemic makes all the healthcare systems as well as the Iranian`s pay serious attention to the areas of procurement, distribution, performance assessment, development, compensation systems and other related human resources management as the prerequisite and necessity [19]. According to the global statistics, more than 160 million cases of confirmed morbidities of COVID-19 and about 3 million related deaths have been reported till May 2021. Moreover, national statistics have reported more than 2 million cases of morbidity and more than 75000 cases of death because of COVID-19 in Iran [20]. Rassouli et al. (2020) have reported many challenges which intensified the mortality and morbidity in the country among them the cultural condition, shortages in the health human resources, lack of Personal Protective Equipment (PPE), and diagnostic and treatment facilities are more highlighted [21]. Considering these conditions and in order to better management of the pandemic in Iran, 41 referral hospitals, 168 hospitals and health centers with the potentiality of emergency evacuation have been allocated to the COVID-19 related services. Meanwhile, more than 5000 urban health stations and 5000 rural comprehensive health centers were considered for tracing and following the suspected and positive cases [22]. All of these have occurred in a condition that the country`s infrastructure has been damaged because of the impacts of long politico-economic sanctions, the restriction of the resources is obvious both in the area of human resources and other physical and financial resources [23].

In such a condition, it is thoroughly clear that optimal management of such facilities depend on effective health human resources management and timeliness identification of the related challenges in this area can pave the way for policymakers toward better management of the pandemic. According to the necessity of access to the related, comprehensive and timeliness knowledge in this field, this study is aimed to explore the challenges of human resources management in healthcare sector during COVID-19 pandemic in Iran. The results can make the deep, customized and applied knowledge for Iranian policymakers and those with the similar settings along with applied recommendations for preparedness against new probable outbreaks.

## Methods

This was a qualitative study conducted in 2020 applying a content analysis approach. The aim of the qualitative study was to achieve the deep understanding of the challenges in the area of health human resources management during COVID-19 in Iran. This methodology was applied for its best compatibility for a systematic deep description of the experiences and viewpoints as well as concept understanding among social organizations. At the same time, we applied content analysis approach for better information management, identification of the challenges and optimal decision making via a mechanism of categorizing, analyzing and determining the concepts for answering the scientific social issues [24].

### Participants

The present study population includes the experts in the area of health human resources management the same as Medical Universities`human resources managers, hospital managers and the health centers and health networks managers. More than the aforementioned official position, at least 2 years of experiences in the area of health human resources management. Snowball sampling was used to include the most experienced and knowledgeable informants. The aim of this purposeful sampling is to include the participants according to the aim of the study rather than accidental methods [25]. These participants were selected from different Medical Universities in north, south, east and the center of the country. Table 1 demonstrates the demographic characteristics of the present participants.

**Table 1:** Frequency distribution of the study participants.

Variables	Category	Number	Percentage
Age (Year)	< 40	9	39.13
	40–50	12	52.17
	> 50	2	8.70
**Total**	**------**	**23**	**100**
Gender	Male	16	69.57
	Female	7	30.43
**Total**	**------**	**23**	**100**
Marital Status	Single	1	4.35
	Married	22	95.65
**Total**	**------**	**23**	**100**
Work experience	< 10	11	47.83
	10–20	10	43.48
	> 20	2	8.69
**Total**	**------**	**23**	**100**
Level of Education	BSc	2	8.70
	MSc	16	69.57
	Ph.D.	5	21.73
**Total**	**------**	**23**	**100**
Position	Health Human Resources Managers	9	39.13
	Governmental Hospitals Or Health Centers`Managers	8	34.78
	Health Network Managers	6	26.09
**Total**	**------**	**23**	**100**
Organization	Tehran University of Medical Sciences	4	17.39
	Golestan University of Medical Sciences	2	8.69
	Shiraz University of Medical Sciences	3	13.05
	Esfahan University of Medical Sciences	3	13.05
	Fasa University of Medical Sciences	1	4.34
	Bandar-e-Abbas University of Medical Sciences	3	13.05
	Zahedan University of Medical Sciences	2	8.69
	Mashhad University of Medical Sciences	3	13.05
	Tabriz University of Medical Sciences	2	8.69
**Total**	**------**	**23**	**100**

### Data collection

An interview guide was applied for conducting semi-structured interviews. Achieving this purpose, one of the researchers (ARY) referred to the human resources managers, hospital managers and health centers and health networks managers and after a formal coordination and prior permission, the semi-structured interviews were conducted.

The interview guide included 10 main questions (Table A in S1 Appendix). In order to achieve a deep understanding of the challenges, probing questions were used to identify how and why. The interviews were started with “how is the status of health human resources management in Iran?” and then directed and continued according to the aims of the study.

The time and place of each interview was flexible and determined according to the tendency and condition of the participants. All the interviews were conducted in Persian and the content of the interview sessions were recorded right after a prior permission of the participants. All the interview content was translated in English at the analysis stage. The interviews were continued to reach saturation level.

### Data analysis

In order to analyze the data, latent content analysis was used. For this purpose, first the researchers listened carefully to all the audio voices several times and all the content was changed to transcript word by word. After transcription, all the written content was read line by line for a comprehensive familiarization with the data. In this step, those phrases or sentences which better indicate the concept according to the aim of the study were highlighted as the meaningful units. After several times reviewing the meaningful units, the related codes were extracted and labeled (Table B in S1 Appendix). This open coding process was continued for the whole text and at the next step, the emerged coded were categorized and integrated in the appropriate categories according to the similarities and differences among their concepts. These were refined as the sub-themes. Then, at the final step, by categorizing the sub-themes, the main themes were emerged, labeled and defined. In order to assure achieving the saturation level at the analysis stage, after conducting each interview, the process of transcription and open coding were carried out.

Meanwhile, in order to assure the validity of the data and robustness of the analysis, four criteria of credibility, transferability, dependability and confirmability suggested by Guba and Lincoln (1989) were applied as follows [26]: credibility was achieved via a prolonged engagement of the researchers in the data collection and data analysis process, at the same time, the interviewers tried to make an effective and trust-based atmosphere for mutual interaction with the participants. Finally, credibility was increased through applying member checking to test the findings and interpretations with the participants. In order to assure transferability, the researchers have tried to provide thick description of the results. Also, an effective and trust-based relationship was built with the participants through clear stating of the study goals and assuring the confidentiality of the data without the probability of identifying each participant. For the third criteria, dependability, the researchers have tried to create a logical, traceable and clear process for the whole research. The comprehensive audit of the research process was used to increase dependability of the study. And finally, to get confirmability of the research, it was important to be able to distinguish among the interpretations of the researchers and the participants`statements. For this purpose, the researcher tried to bracket their previous assumptions in the process of collecting and analyzing the data [27].

The participants were also assured to have a right for quitting the interview process in every stage that they want to stop talking. Data were analyzed applying MAXQDA_10_ software by the researchers with no conflict of interest with the topic and sufficient reflexivity with the qualitative analysis.

### Ethics statement

This study is approved by Shiraz University of Medical Sciences ethics committee with the approval number of IR-SUMS-REC-855-1399. The written informed consent form was obtained from all the participants.

## Results

Among 23 interviewees, 9 participants were health human resources managers in one of the Iranian Medical Universities, 8 participants were among one of the governmental hospitals or health centers`managers and 6 of them worked as health network managers. 16 participants were male and 7 were female (Table 1).

The content analysis of the interviews led to explore three main themes as the main challenges of health human resources management challenges during COVID-19 pandemic in Iran. These main themes were: the organizational challenges, the legal challenges and the personal challenges (Table 2). The legal challenges mostly derived from the superior levels than the organization such as government, Ministry of Health, and management and planning organization that all act as the supervisors in the country. The organizational challenges are focused on those problems that are related to the organizations. Here we mean the university of medical sciences, the affiliated hospitals and health centers by the term organization. And finally, the personnel challenges consider the problems at the individual level. The relate concept of the aforementioned themes and their related sub-themes subordinating with the related quotations are as follows:

**Table 2:** The challenges of health human resources management during COVID-19.

Main themes	Sub themes
**Organizational challenges**	Restricted financial resources
	Compensation discrimination
	Discrimination in staffing distinction points and privileges
	Imbalance in the workload
	Weak organizational coordination
	Inefficient inter-sectoral relationships
	Conflicted and parallel decisions
	Inefficient distribution of the human resources
	lack of applied, planed and integrated education
	Lack of integrated health protocols for the personnel`s health
	Lack of appropriate evaluation of performance
	High employee turnover
	Shortage of specialized manpower
**Legal challenges**	Lack of continuous supportive services for sick personnel
	Inappropriate approaches and lack of definite instruction for teleworking
	Lack of alternative plans and regulations instead of the missed human resources
	Lack of clear approaches and protocols for staffing
**Personal challenges**	Insufficient knowledge of the employees about COVID-19
	psychological disorders among personnel
	Reduction of self-confidence and self-esteem among personnel
	Personnel`s burnout
	Workload increase and personnel`s fatigue
	Reduced level of job satisfaction, incentives and moral sense
	Effects of colleague and patients`bereavement
	Unsafety sense against the work place

### Organizational challenges

According to Table 2, the organizational challenges contain 13 sub-themes that are described as follows:

Restricted financial resources for the personnel`s compensation is among one of the organizational challenges for health human resources managers. One of the participants said:

“*The hospitals`allocative revenue was decreased because of the reduction of non-infectious admissions and elective surgeries cancellations*. *Now we face a severe lack of financial resources to compensate the personnel`s services” [P*_*7*_*]*.

Another participant stated to the restricted financial resources as follows:

“*In spite of financial supports from the Ministry of Health*, *such these funds aren’t sufficient for COVID-19 costs particularly the costs of personnel compensation” [P*_*16*_*]*.

Another subtheme in this area was compensation discrimination and inequality in the personnel`s payments particularly according to their type and level of specialty. One participant said:

“*The payments of the personnel specialty those working in the infectious wards with COVID-19*, *are not acceptable*. *These payments are also being allocated and distributed equally and on the base of the personnel`s specialty” [P*_*3*_*]*.

Another participant believed that:

“*The rewards allocated to the personnel who worked in COVID-19 wards are not determined on a scientific and clear basis*. *For instance*, *a physician who is working in a diagnostic referral hospital may receive a different amount comparing another physician in a supportive center*. *It is not fair*…*” [P*_*20*_*]*.

Another challenge related to the organizations was an imbalance in the workload among different specialties. One participant said:

“*COVID-19 may result in increasing the workload among personnel with infectious and internal specialties while decreasing among some other specialties” [P*_*9*_*]*.

Another participant added:

“*During COVID-19 outbreak*, *the need for some services increased like the services of allied workers in nursing” [P*_*13*_*]*.

The weak inter-sectoral and intra-sectoral coordination, ineffective communications, parallel and conflicted decision making are among other health human resources management challenges. In this area a participant said:

“*there is not an effective communication and coordination for planning and management in and among the universities of medical sciences and also between the superior organizations the same as the Ministry of health and the medical universities” [P*_*4*_*]*.

Another participant added:

“*The coordination in the area of health human resources management during COVID-19 is poor even inside the hospitals and health centers of a medical university*. *For instance*, *for teleworking of the personnel each area has its own protocol while there is a mutual coordination is really necessary*. *Also*, *for procurement and transferring the personnel*, *there is no unique and integrated protocol and it is not obvious that who has the responsibility in this regard “[P*_*12*_*]*.

Another participant believed that:

“*There is no defined approach for applying the previous knowledge and experiences of the other medical universities about management of the health human resources during the pandemic“[P*_*5*_*]*.

Lack of documented, integrated and pre-planned training courses to empower the personnel with updated knowledge during the pandemic was among other main organizational themes. One participant mentioned that:

“*Those training courses with the aim of health protocols for the personnel are not directed in a determined way*. *In another word*, *every other day some educational programs are held without any prior information or planning*!*” [P*_*22*_*]*.

Lack of possibility for appropriate and exact performance evaluating of the personnel were among other organizational challenges. Another participant believed that:

“*There is no appropriate supervision on the personnel`s performance during teleworking*. *This restricts the issue of performance assessment of the human resources” [P*_*14*_*]*.

The other subtheme related to organizational challenges was the high demand for employee turnover. While the tangible shortage for human resources was felt particularly the skillful specialized personnel. Moreover, lack of integrated documented programs for procurement and replacement of the required personnel was among other challenges. Another participant declared:

“*During the pandemic and because of the lack of adequate understanding about the nature of the disease and the fear of being sick*, *many personnel specially those employed in the hospitals requested to change their working place or even quit their job” [P*_*6*_*]*.

Another participant explained:

“*During COVID-19 pandemic*, *there was no documented and integrated program for procurement of the physicians and staffing” [P*_*23*_*]*.

And finally, the last subtheme in this area was related to discrimination in staffing distinction points and privileges. One participant said:

“*Staffing privilege is not fair and equitable*. *For instance*, *those personnel who have been working in the hospitals from the beginning of the pandemic are considered the same as those who have already started their jobs in the hospitals*!*” [P*_*19*_*]*.

### Legal challenges

Lack of infrastructures and appropriate approaches and instructions for teleworking was among one of the legal challenges. From the interviewees`opinion there was no definite unique protocol for tele-working from the government. Such a condition can make disorder and dissatisfaction among the personnel. In this area a participant complained that:

“*when the aim of teleworking is the reduction or maybe cutting the transformation chain of the disease*, *it should be considered that procuring the infrastructures of communication and information technology is necessary… it is such a thing that no unique instruction is available for…” [P*_*17*_*]*.

Lack of continuous supportive services for sick personnel along with lack of applied protocols for the personnel`s safety and health during the pandemic such as providing supportive and palliative cares were among the other identified legal challenges. In this regard a participant said:

“*Although supportive and palliative cares for sick personnel were started well at the beginning of the pandemic*, *during the time*, *the integrity and the quality of the services missed*. *Now*, *the main concentration and attention is on the patients and those of the personnel are not in an acceptable level” [P18]*.

Lack of alternative plans and regulations instead of the missed human resources was achieved as another main theme. It seems that there is no unique regulation for procurement of professional health personnel who may die, miss or become disabled as a result of pandemic. One participant said:

“*Unfortunately*, *we lose many of our personnel specially in the hospitals but there is no definite protocol how we can recruit new staff instead…”[P20]*.

### Personal challenge

The third category of challenges that the present participants have experienced them were related to the personal challenges. In this area, insufficient knowledge of the employees about COVID-19 was mentioned. One of the interviewees said:

“*One of the most important actions for managing the COVID-19 pandemic was the attempts for improving the level of knowledge and information of the personnel to be able for preventing the disease*. *While some personnel were not aware of the prevention methods and the ways of transferring the virus*. *For instance*, *we see such behaviors that some of the employees are having meals close to each other during their work hours*!*” [P*_*5*_*]*.

Another participant added:

“*There are some behaviors in the work places that reflect the insufficient information of the personnel against health preventive protocols of COVID-19” [P*_*3*_*]*.

Psychologic disorders among personnel and reduction of self-confidence and self-esteem among them, burnout, increase in the personnel`s load work, fatigue and reduction in their level of job satisfaction, incentives and moral sense are among other subthemes in this category. One of the participants stated that:

“*COVID-19 can lead to many physical*, *psychological and social effects on the personnel*… *When talking to them*, *it is obviously observable that they suffer stress*, *anxiety and sometimes depression” [P*_*11*_*]*.

Another participant added:

“*COVID-19 results in an extreme workload and psychological problems the same as fear and anxiety specially among those employees who worked directly with the infected patients” [P*_*3*_*]*.

Or, another participant said elsewhere:

“*working in such a condition is accompanied with stress*, *anxiety and tensions*. *This situation can lead to decreasing in the level of incentives and job satisfaction among the personnel and affect their self- confidence” [P*_*1*_*]*.

About the increasing workload, fatigue and burnout, one of the participant mentioned:

“*one of the challenges in front of the human resources management is the increase in the personnel`s workload particularly among those who work directly with the infected patients*. *such a workload can result in fatigue and complains among physicians and the nurses” [P*_*3*_*]*.

Another interviewee claimed that:

“*COVID-19 has led to a severe workload on the healthcare personnel*. *Such a pressure may cause burnout” [P*_*15*_*]*.

Finally, the last subtheme related to the personal challenges was the uncertainty and unsafety sense against the work place. In this regard, a participant said:

“*Health workers cannot be certain about the probability of being infected*. *They are always engaged in sterilizing their personal equipment the same as table*, *chair*, *telephone and so on*. *This can lead to obsessive-compulsive disorder among these personnel” [P*_*1*_*]*.

**Fig 1:**
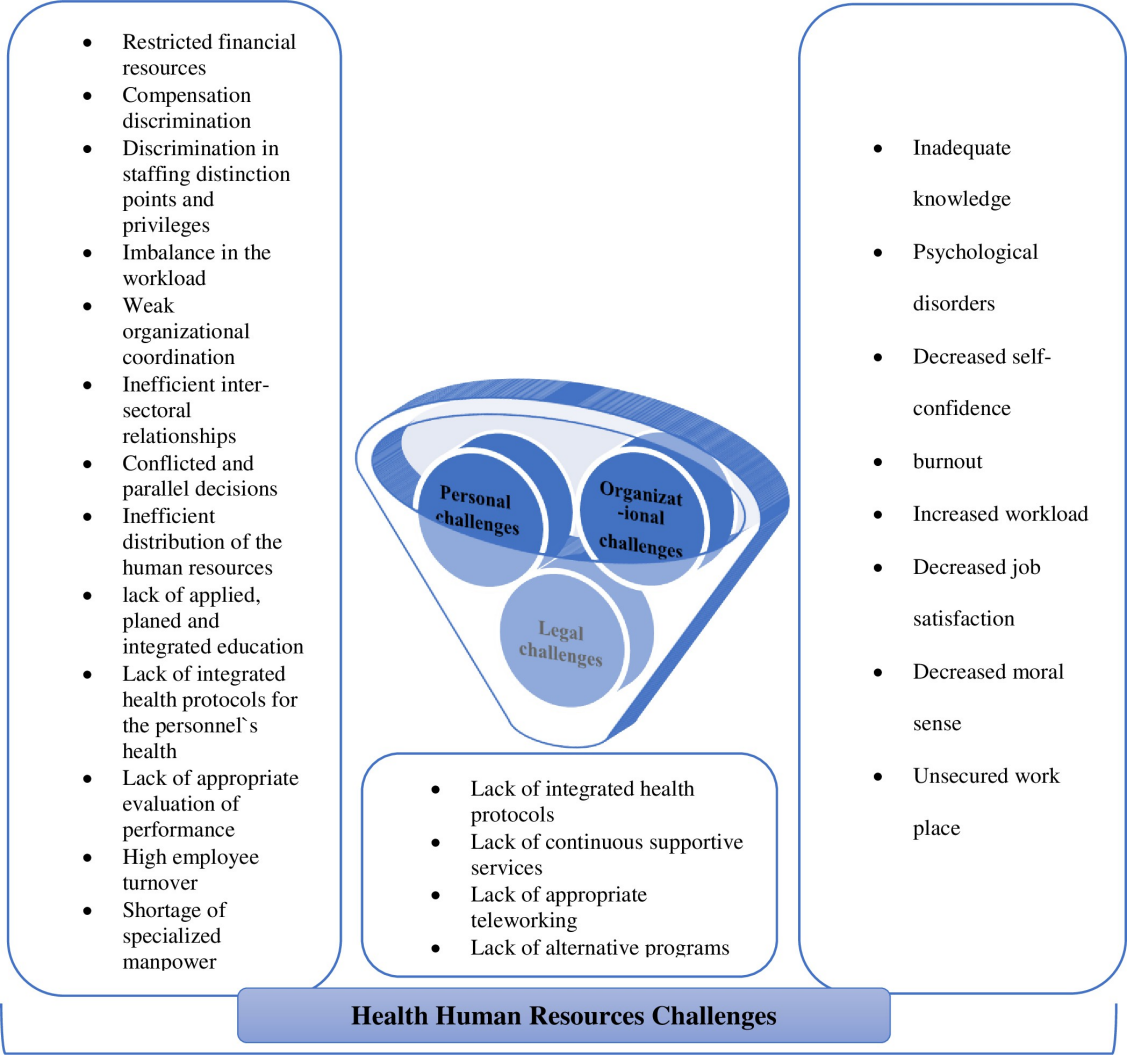
The schematic network of Iranian health human resources challenges during COVID-19.

For better understanding of the challenges, Fig 1 simply illustrates the challenges of health human resources management during COVID-19 in Iran.

## Discussion

The present study is conducted to explore the challenges of health human resources management during COVID-19 in Iran as one of the high rate infected regions. The present analysis has explored three main categories of challenges related to the organization and the own personnel, as well as the legal challenges each with 13, 4 and 8 subthemes respectively. For better discussion and interpretation of the findings we have gone through them via separate sub-headings.

### Personal challenges

Eight sub-themes were categorized to demonstrate personal challenges as follows: insufficient knowledge, psychological disorders, reduction of self-confidence and self-esteem, burnout, fatigue and workload, Reduced level of job satisfaction, incentives and moral sense, effects of colleague and patients`bereavement and unsafety sense against the work place.

### Insufficient knowledge of the employees about COVID-19

The present results show that insufficient knowledge about COVID-19 was considered as a challenge of health human resources. The unknown nature of disease, type of treatment, disease prognosis, the predication of prevalence speed, the probability of the recurrence, unknown transmission ways and undefined clinical manifestations are among ambiguous reasons and insufficient knowledge specially at the beginning of the pandemic [28, 29].

According to the related studies at the beginning of the pandemic, a considerable amount of health workers benefits from a poor knowledge of transmission ways of the disease and the initial clinical symptoms [30, 31]. According to Fathi et al. (2020), shortage of specialized knowledge, insufficient preparedness and lack of access to applied skills against managing and controlling the disease were among the healthcare workers`challenges during COVID-19 [29]. Lack of knowledge and experience were announced as one of the stressful factors among health workers during COVID-19 pandemic [32]. Considering that health workers are providing services on the front line of the health systems against COVID-19, inadequate knowledge and wrong attitudes among them can directly affect their behaviors and lead to delays on disease diagnosis, poor performance of infection control and disease spread [33].

### Psychological disorders among personnel

Psychologic disorders among healthcare workers were among the other identified challenges in the area of personal challenges. According to the other results, those workers who are directly engaged in COVID-19 wards have experienced many psychological challenges because of unpredictable conditions, high workload, unknown nature of the disease, frequent changes of the protocols and rapid changes in the policies, information and operational roles, great fatigue, high rate of mortality among the patients, fear of being infected, lack of supportive psychological, social and organizational packages and also lack of access to personal protective equipment (PPE) [28, 29, 34–39].

Results of a review study shows that during COVID-19 pandemic, negative psychological effects the same as stress, depression, anxiety, insomnia and sense of anger were increased among healthcare workers and other engaged people with COVID-19 significantly [32]. The experience of mental derangement and negative emotions was one of the main challenges in front of the physicians and nurses who are working in the covid-19 Intensive Care Units (ICU) in Qom, Iran [29]. In another study in Canada, mental disorders were considered as the main challenge of the nurses against the initial response to COVID-19 [38]. Applying mental health experts for enabling healthcare workers during disaster, empowering the personnel`s skills in stress management, applying incentives and physical and spiritual supports along with providing necessary information about the pandemic can help decreasing the mental disorders.

### Reduction of self-confidence and self-esteem among personnel

In the area of personal challenges, the present results have also led to decrease in self-confidence and self-esteem of the healthcare workers. Lak of knowledge, experience and skills can be a reason for low self-confidence. According to the other results, unknown nature of COVID-19 may cause the healthcare workers suffer from low self-confidence for service delivery, lack of knowledge and experiences in the ICUs have also led to self-confidence reduction of the personnel. Inability in service delivery is also caused to the sense of unsafety and lack of self-confidence even in the most experienced nurses [40, 41]. Against the aforementioned studies, in a conducted study in Iran, some of the participants claim that they like the challenges and the routine tasks cannot satisfy them, so overcoming the challenges can lead to their self-confidence increase [39]. In another qualitative study in Italy, the nurses believe that in spite of the community`s gratitude, some people`s behaviors are not gracious and the inappropriate reactions may lead to the sense of worthlessness among the nurses. Such these senses can influence the personnel`s self-esteem negatively [36]. Unpleasant experiences and worthlessness because of the inappropriate behaviors and reactions of the community are also reported among the Iranian nurses, too [39]. In spite of the present results, some of the Iranian physicians and nurses expressed a deep sense of love to their profession and emphasized on the senses of proud and worth, while helping the others [34, 39]. Some Italian and Chinese nurses have also reported a sense of value and proud to provide services to the patients infected with COVID-19 [36, 41].

### Reduced level of job satisfaction, incentives and moral sense

The reduction in the level of job satisfaction was among another challenges in the area of the personal challenges. Results of a qualitative study in Jordan shows that the irrational and imposed actions and decisions, dissemination the misinformation and unclear statements from the government can affect the physicians`job satisfaction. Other factors like inadequate number of the health workers, lack of integrated protocols and, fatigue as a result of work overload have also influenced the job satisfaction negatively [42]. Job dissatisfaction can also be considered as a main factor of employee turnover. In a study conducted in Iran, some types of insecurity, ambiguity and high tendency of quitting the job were reported among the nurses`personal lives who worked in in the ICUs of COVID-19 patients [28]. In Italy, the nurses have experienced job dissatisfaction regarding the severe difficulties from the beginning of COVID-19 [36]. But another study in Iran, the nurses have claimed that they are happy and satisfied because of the potentiality of helping the others [39].

### Effects of colleague and patients`bereavement

Demoralized of the personnel because of their colleagues and patients`deaths was among another challenges in the personal area. Other studies`results have also shown that the sense of sorrow, depression and demoralized after the sudden death of the patients were experienced by almost all the health workers. They also reported the sense of inefficacy and guiltiness because of any alternative and solutions for saving the patients`lives [29, 38, 40, 41, 43, 44]. Alrawashdeh et al. (2021) have also reported that the increase in the number of mortality cases in Jordan was among the factors that lead to experiencing of sorrow among the physicians [42]. The nurses also, feel sad because of the pains and discomfort of their collegues during being infected by COVID-19 [40]. According too another study, the nurses have reported that they are afraid to make their collegues sick and try to protect each other [36].

### Personnel`s burnout and workload and fatigue

Another personal challenge was the increase in the personnel`s workload, fatigue and burnout. Factors like working in the overcrowded hospitals, long work hours, night shifts, lack of inadequate access to PPE, personnel shortages, the collegues illness or death, unstopping work without leaves or vaccations and the difficulties of working with protective sheilds, cloths and sanitation materials have positive relationship with the working burnout in the other studies [28, 29, 41, 42, 45]. Applying PPEs has been complicated the service delivery process and cased physical problems like dehydration, urine infection, constipation, nose and face hurt and injuries and the feeling of asthma [28, 45, 46]. According to Fontanini et al. (2021), the negative feelings like sorrow, fear, inability, anxiety and anger played an important roles among the nurses [36]. Roslan et al. [47] have also reported the negative physical, psychological and social affects related to job burnout during COVID-19 pandemic that were experienced by almost half of the health workers [47].

### Unsafety sense against the work place

The last challenge in this area was unsafety sense against the work place. The risk of being infected by the virus and morbidity because of the shortages of PPEs particularly at the beginning of the pandemic was reported in many studies [28, 29, 36, 40, 41, 45, 48]. Such a condition causes great concerns of transferring virus to the health workers`families. It is against the fact that health care workers should work in a completely safe and secure places to have enough concentration for service delivery. Sufficient requirements and facilities are the most vital elements to make such safe places for the personnel [49].

### Organizational challenges

Thirteen sub-themes were categorized to develop organizational challenges as follows: restricted financial resources, discrimination in compensation and utilizing points and privileges, imbalance in the workload, weak organizational coordination, inefficient inter-sectoral relationships, conflicted and parallel decisions, inefficient distribution of the human resources, lack of applied, planed and integrated education and protocols for the personnel`s health, lack of Lack appropriate evaluation of performance, high employee turnover, and shortage of specialized manpower.

### Restricted financial resources

One of the challenge in this area was the restricted financial resources for the personnel`s compensation. The public governmental hospitals have encountered many problems that lead to reducing their financial potentiality the same as increasing average length of stay, cancelling elective surgeries, providing PPEs and supportive facilities for the personnel and the costs of public education of the community [49]. Other financial challenges were reported for the health systems during COVID-19 pandemic the same as increase in costs and at the same time revenue reduction [48, 50]. Analysis of the interviews with the hospital managers of Colombia have shown that via cost increase and the loss of revenues during COVID-19, many hospitals have forced to fire their personnel [48]. An Iranian study also showed that the nurses have complained of the delay for their services compensation and the little payments they have received comparing their huge unbearable tasks [28].

### Compensation discrimination

Compensation discrimination particularly among different specialties was among other challenges in this area. According to the present results, the organizational inefficiency in supporting the nurses was among the concepts extracted from the interviews. They expect that their chief supervisors visit their work fields and try to improve their incentives [28]. Other nurses have complained that they have not considered by their managers [36, 37]. Iranian nurses have specifically pointed to the discrimination imposed on them comparing the physicians in the same wars. They thought such senses of discrimination and injustice can decrease their incentives for service delivery in a great deal [28]. In this regard it should be considered that although the financial incentives can be an approach for increasing job satisfaction, in such a pandemic situation, psychological, moral and spiritual supports should not be neglected. The sense of sympathy and mental support can greatly apply for appreciating the personnel`s hard works.

### Imbalance in the workload

Another related challenge in this area was imbalance in the workload among different specialties. According to the Colombian hospital managers, during COVID-19 pandemic, the hospitals suffer from specialized personnel for service delivery. Such a problem has led to increase in the personnel workload in a way that, the managers was concerned the employee turnover as a result of their workload [48]. The increase in the hospital workers is reported in another study that led to quitting the workplace by some of the personnel particularly at the beginning the pandemic [44]. In another study, a severe conflict was perceived by the personnel who worked in the ICUs. In one hand, they had a sense of sympathy to the patients and in the other hand, they felt injustice comparing their tasks with the others [29].

### Weak organizational coordination

Weak organizational coordination, inefficient inter-sectoral relationships and conflicted and parallel decisions were among other challenges in this area. According to the present results, the coordination and management of health human resources were poor among different sectors of the medical universities and the supervisory organizations during COVID-19. Some of these problems can be caused by the complex nature of the health systems. For instance, in a qualitative study, the participants declare that the cooperation, coordination and teamwork were not occurred appropriately among healthcare members the same as family physicians [51].

### Discrimination in staffing distinction points and privileges

Discrimination in staffing distinction points and privileges was another organizational challenge. In this regard, the Iranian Ministry of Health was approved to give 20 extra points for the employment of those health workers who have the experience of voluntary cooperation with the hospitals during pandemic [52]. According to an opinion survey, the participants claimed that the staffing points and privileges were not justice and fair play. Also the centralized process of staffing by the medical universities without considering the hospitals favorite criteria and needs were considered as the main challenges by the studies [18].

### Lack of applied, planed and integrated education

Among other organizational challenges, we can refer to lack of applied, planed and integrated education. While the continuous training for the professional personnel is very significant during the pandemic because of the new and indefinite nature of the pandemic, the present results show that a documented approved educational curriculum was not available for the personnel. Similarly, Arnetz et al. (2020) have emphasized that the presented trainings for those personnel who were directly engaged with COVID-19 were weak in the USA [43].

### High employee turnover and shortage of specialized manpower

Other results have mentioned high employee turnover, lack of clear approaches for staffing and, shortage of specialized manpower as the other organizational challenges. The shortage of health human resources was among the reported challenges all over the world during pandemic. The increased workload, employee turnover, staff firing because of the hospitals`financial problems and missing the healthcare workers because of their illness or death were the main reasons for this shortage [28, 29, 36, 39, 41, 48]. Alrawashdeh et al. (2021) in Jordan have announced conflicted decisions in the hospitals along with lack of experiences of the managers for estimating the necessary healthcare workers, as the main resons of shortages [42]. Iranian policymakers have tried to apply alternative solutions for overcoming the barriers of healthcare workers via the use of vulentery people and groups to control the disaster caused by COVID-19 pandemic [53]. Such policies can organize the potentiality of the specialized vulenteers and lead to the community advocacy in order to manage the shortage of healthcare workers.

### Lack of appropriate evaluation of performance

Lack of appropriate evaluation of performance were among other organizational challenges. This challenge can be considered both for in site working and the teleworking conditions. According to the results of a study, the managers`viewpoints showed that the possibility of the exact performance of the personnel and their work progression assessment may face many challenges during teleworking [54].

### Legal challenges

Legal challenges include four sub-themes of: lack of continuous supportive services for sick personnel, inappropriate approaches and lack of definite instruction for teleworking, lack of alternative plans and regulations instead of the missed human resources, and lack of clear approaches and protocols for staffing.

### Inappropriate approaches and lack of definite instruction for teleworking

Inappropriate approaches and instructions for teleworking was among the legal challenges. In this area, lack of unique protocol containing the circumstances and requirements for teleworking was considered as a significant issue. The supervision methods during the personnel`s teleworking along with lack of communication infrastructures are considered as the main challenges of teleworking [55]. Results of another qualitative study have also shown that those personnel who have experienced teleworking during COVID-19 pandemic, felt that when missing the condition of their working place via teleworking, the possibility of receiving the feedbacks and idea sharing with the team members and supervisors were missed [56].

### Lack of continuous supportive services for sick personnel

Lack of integrated unique health protocols and instructions for the personnel`s health and lack of continuous supportive services for sick personnel during COVID-19 pandemic were among the identifies sub-themes in the present study. According to a qualitative study conducted in UK, the protocols of applying the PPEs were continuously changed that can lead to ambiguity and lack of trust among healthcare workers [45]. Colombian hospital managers have also emphasized that conflicted and reverse guidelines can make the healthcare workers confused [48]. Catania et al. (2021) have also considered the implementing of a unique protocol about applying the PPEs as one of the experienced challenges by the healthcare workers in Italy [40]. Crowe et al. (2021) have also stated that changes in the PPE protocols make many conflicts for the healthcare workers. For instanse the personnel were concerned that whether thay can be aware of all the changes announced for PPE protocols in the hospitals or not [38].

Considering all discussed above, it would be significant for the policymakers to formulate related applied policies and practises according to the identified challenges and the similar findings of the other studies. Results of this qualitative study can pave the way for better managing of the health human resources during the pandemic after seeking the applied interventions for overcoming and decreasing the above challenges.

## Conclusion

Health human resources management during COVID-19 pandemic has faced three categories of challenges. Although the legal challenges are beyond the managers`authority, their identification can open a new window for health policymakers to move toward correcting the legal deficiencies and making integrated and unique instructions, regulations and protocols for those organizations with the same conditions. Organizational and personal challenges are among two other main challenges identified in the present study. Serious attention to these challenges should be considered by health policymakers in order to be prepared for facing new probable outbreaks and managing the present condition. The integrated comprehensive planning of human resources management for COVID-19 along with supportive packages for the personnel can be helpful. It is recommended to the health human resources policymakers to plan a comprehensive roadmap for staffing, educating, developing and improving the healthcare workers for near future.

## Supporting information

S1 File (DOCX)

S1 Appendix The interview guide in original language and its translation in English. (DOCX)
