# Supplementary material for: Health human resources challenges during COVID-19 pandemic; evidence of a qualitative study in a developing country
Source: PLoS One. 2022 Jan 24;17(1):e0262887. doi: 10.1371/journal.pone.0262887 (PMC8786119; doi:10.1371/journal.pone.0262887)
Supplement: S1 File — (DOCX) [file pone.0262887.s001.docx]

**فرم سوالات مصاحبه طرح پژوهشی با عنوان:**

چالش‌های مدیریت منابع انسانی بخش سلامت طی بحران کرونا ویروس (کووید-19):

شواهد یک مطالعه کیفی از کشور در حال توسعه

| ردیف | سوالات مصاحبه |
| --- | --- |
| 1 | وضعیت مدیریت منابع انسانی بخش سلامت در کشور ایران در بحران کووید-19 به چه صورت است؟ |
| 2 | بیماری کروناویروس (کووید-19) چه تاثیری بر مدیریت منابع انسانی سلامت در کشور ایران داشته است؟ |
| 3 | طی پاندمی کووید-19 مدیریت منابع انسانی نظام سلامت کشور ایران با چه چالش های در حوزه برنامه‌ریزی منابع انسانی روبرو است؟ |
| 4 | طی پاندمی کووید-19 مدیریت منابع انسانی نظام سلامت کشور ایران با چه چالش های در حوزه تأمین منابع انسانی روبرو است؟ |
| 5 | طی پاندمی کووید-19 مدیریت منابع انسانی نظام سلامت کشور ایران با چه چالش های در حوزه گزینش و استخدام نیروی انسانی روبرو است؟ |
| 6 | طی پاندمی کووید-19 مدیریت منابع انسانی نظام سلامت کشور ایران با چه چالش های در حوزه تخصیص نیروی انسانی روبرو است؟ |
| 7 | طی پاندمی کووید-19 مدیریت منابع انسانی نظام سلامت کشور ایران با چه چالش های در حوزه آموزش نیروی انسانی روبرو است؟ |
| 8 | طی پاندمی کووید-19 مدیریت منابع انسانی نظام سلامت کشور ایران با چه چالش های در حوزه ارزیابی و ارزشیابی عملکرد نیروی انسانی روبرو است؟ |
| 9 | طی پاندمی کووید-19 مدیریت منابع انسانی نظام سلامت کشور ایران با چه چالش های در حوزه جبران خدمات نیروی انسانی روبرو است؟ |
| 10 | طی پاندمی کووید-19 مدیریت منابع انسانی نظام سلامت کشور ایران با چه چالش های در حوزه امنیت و سلامت جسم و روان منابع انسانی روبرو است؟ |

- **سایر سوالات بر اساس روند پیشرفت مصاحبه: ..............**

| Would you please describe the status of health human resources management in Iran during COVID-19 pandemic? |
| --- |
| What do you think that how COVID-19 affects the Iranian health human resources management? |
| In your opinion, during the pandemic what are the main challenges of Iranian health human resources management in the area of planning? |
| In your opinion, during the pandemic what are the main challenges of Iranian health human resources management in the area of personnel procurement? |
| In your opinion, during the pandemic what are the main challenges of Iranian health human resources management in the area of personnel recruitment and staffing? |
| In your opinion, during the pandemic what are the main challenges of Iranian health human resources management in the area of personnel allocation? |
| In your opinion, during the pandemic what are the main challenges of Iranian health human resources management in the area of training and education? |
| In your opinion, during the pandemic what are the main challenges of Iranian health human resources management in the area of performance assessment? |
| In your opinion, during the pandemic what are the main challenges of Iranian health human resources management in the area of personnel compensation? |
| In your opinion, during the pandemic what are the main challenges of Iranian health human resources management in the area of personnel safety and health? |
