# Supplementary material for: Health human resources challenges during COVID-19 pandemic; evidence of a qualitative study in a developing country
Source: PLoS One. 2022 Jan 24;17(1):e0262887. doi: 10.1371/journal.pone.0262887 (PMC8786119; doi:10.1371/journal.pone.0262887)
Supplement: S1 Appendix — (DOCX) [file pone.0262887.s002.docx]

**S1 Appendix**

**Table A- The interview guide of the study**

| Would you please describe the status of health human resources management in Iran during COVID-19 pandemic? |
| --- |
| What do you think that how COVID-19 affects the Iranian health human resources management? |
| In your opinion, during the pandemic what are the main challenges of Iranian health human resources management in the area of planning? |
| In your opinion, during the pandemic what are the main challenges of Iranian health human resources management in the area of personnel procurement? |
| In your opinion, during the pandemic what are the main challenges of Iranian health human resources management in the area of personnel recruitment and staffing? |
| In your opinion, during the pandemic what are the main challenges of Iranian health human resources management in the area of personnel allocation? |
| In your opinion, during the pandemic what are the main challenges of Iranian health human resources management in the area of training and education? |
| In your opinion, during the pandemic what are the main challenges of Iranian health human resources management in the area of performance assessment? |
| In your opinion, during the pandemic what are the main challenges of Iranian health human resources management in the area of personnel compensation? |
| In your opinion, during the pandemic what are the main challenges of Iranian health human resources management in the area of personnel safety and health? |

**Table B- The final open codes achieved from the interviews**

| Lack of financial resources, lack of credit, shortage of cash, budget deficiency |
| --- |
| Discrimination in payments, unfair compensation, unfair salaries |
| Raw and non-expert points and privileges for staffing, inequality in staffing, non-exact and scientific scales |
| High workload, high pressure of work, high volume of duties, repeated shifts, long shifts |
| Insufficient planning for workforce, weak communication in human resources management, ineffective relationship in human resource planning, Inappropriate collaboration and advocacy |
| Conflicted decisions, Fragmented decisions |
| Inequality in distribution of the personnel, inequality in human resource allocation |
| Lack of virtual learning, non-integrated education, low and primary level of the courses, |
| Lack of Personal Protective Equipment protocol, lack of personal health instruction, lack of self-protected instructions, indefinite protocol of self-care |
| Non-integrated services to sick personnel, non-continuous care for sick personnel, ineffective support of the sick personnel, vague plans for palliative and supportive care |
| Teleworking full of chaos, lack of utilizing the potentiality of teleworking for some personnel, lack of supervision on teleworking, delay in duties during teleworking, injustice in utilizing teleworking |
| Inappropriate performance assessment, weak process of performance assessment, low quality performance assessment, superficial performance assessment |
| Absence, quitting the job, turnover, transformation |
| Lack of unique plan for personnel recruitment, lack of definite plan for staffing and organizing the personnel, weak mechanism of selecting the workforce |
| Low-skilled workforce, non-empowered personnel, personnel with low level performance, not skilled and expertise personnel. |
| Lack of written regulation for staffing instead of missed personnel |
| Lack of knowledge, lack of skills, lack of information, lack of professional experience |
| Stress, anxiety, depression, mental disorders, mental problems among the personnel |
| Lack of self-confidence, low self-confidence, low self-esteem, lack of self-esteem |
| Fatigue, exhausted personnel, burnout, tiered personnel |
| Low level of job satisfaction, inappropriate satisfaction, lack of moral and incentives |
| Demoralized of the personnel because of their colleagues and patient’s deaths, effects of colleague bereavement, mental disorders because of high mortality rate among patients |
| Obsessive-compulsive state, unsterilized and contaminated working environment, wrong perceptions about the working environment`s health and safety |
